# Vestibular Function Tests for Vestibular Migraine: Clinical Implication of Video Head Impulse and Caloric Tests

**DOI:** 10.3389/fneur.2016.00166

**Published:** 2016-09-30

**Authors:** Woo Seok Kang, Sang Hun Lee, Chan Joo Yang, Joong Ho Ahn, Jong Woo Chung, Hong Ju Park

**Affiliations:** ^1^Department of Otolaryngology, Asan Medical Center, University of Ulsan College of Medicine, Seoul, South Korea

**Keywords:** caloric test, head impulse test, prognosis, sensory organization test, vestibular-evoked myogenic potential, vestibular migraine

## Abstract

Vestibular migraine (VM) is one of the most common causes of episodic vertigo. We reviewed the results of multiple vestibular function tests in a cohort of VM patients who were diagnosed with VM according to the diagnostic criteria of the Barany Society and the International Headache Society and assessed the efficacy of each for predicting the prognosis in VM patients. A retrospective chart analysis was performed on 81 VM patients at a tertiary care center from June 2014 to July 2015. Patients were assessed by the video head impulse test (vHIT), caloric test, vestibular-evoked myogenic potentials (VEMPs), and sensory organization test (SOT) at the initial visit and then evaluated for symptomatic improvement after 6 months. Complete response (CR) was defined as no need for continued medication, partial response (PR) as improved symptoms but need for continued medication, and no response (NR) as no symptomatic improvement and requiring increased dosage or change in medications. At the initial evaluation, 9 of 81 patients (11%) exhibited abnormal vHIT results, 14 of 73 (19%) exhibited abnormal caloric test results, 25 of 65 (38%) exhibited abnormal SOT results, 8 of 75 (11%) exhibited abnormal cervical VEMP results, and 20 of 75 (27%) exhibited abnormal ocular VEMP results. Six months later, 63 of 81 patients (78%) no longer required medication (CR), while 18 (22%) still required medication, including 7 PR and 11 NR patients. Abnormal vHIT gain and abnormal caloric results were significantly related to the necessity for continued medication at 6-month follow-up (OR = 5.67 and 4.36, respectively). Abnormal vHIT and caloric test results revealed semicircular canal dysfunction in VM patients and predicted prolonged preventive medication requirement. These results suggest that peripheral vestibular abnormalities are closely related to the development of vertigo in VM patients.

## Introduction

Migraine is a common disorder characterized by recurrent throbbing headache, most often one-sided, and it is occasionally preceded by aura. An association between vestibular dysfunction and migraine has been proposed and actively investigated (1–3). As a result of such studies, vestibular migraine (VM) is now widely accepted as a unique disease entity (4), although the pathophysiology remains uncertain. VM presents as recurrent episodes of vertigo temporally related to migraine. The lifetime prevalence of VM was found to be approximately 1% in the German population (5). VM afflicts more people than other vertiginous disorders such as Meniere’s disease, vestibular neuritis, and benign paroxysmal positional vertigo (6).

There has been an effort to characterize the disease entity of VM and define the diagnostic criteria for it. Recently, the Barany Society and the International Headache Society released the diagnostic criteria of VM (4). According to it, VM patients should have at least five vestibular attacks. They should have history of migraine with or without aura and/or migraine features with at least 50% of the vestibular episodes. And the vestibular episodes cannot be better accounted for by another vestibular or ICHD diagnosis.

While some studies have assessed the results of vestibular function tests in VM patients (7–12), few have examined the associations between vestibular function test results and subsequent symptomatic improvement on medication. One study revealed abnormal caloric test results in 22% of VM patients and video head impulse test (vHIT) abnormalities in 9% of patients (10), while another revealed abnormal caloric test results in 42% and abnormal vHIT results in 8% of VM patients (11).

In the present study, we analyzed the results of multiple vestibular function tests at baseline, caloric test, vHIT, vestibular-evoked myogenic potential (VEMP), and sensory organization test (SOT) in VM patients and assessed the association of each with subsequent complete response (CR), partial response (PR), and no response (NR) to medication.

## Materials and Methods

Between June 2014 and July 2015, a retrospective chart review identified 81 VM patients according to the diagnostic criteria of the Barany Society and the International Headache Society (4). Patients were examined by the caloric test, vHIT, VEMP, and SOT at the initial visit.

Caloric test and SOT were performed in the same way as previous reports (11, 13). The bithermal caloric test was used, and eye movements were recorded by means of a video-based system (ICS water caloric stimulator NCI-480; Otometrics, Denmark). Each ear was irrigated with a constant flow of water at temperatures at 30 and 44°C for 30 s. The maximum slow-phase eye velocities of the nystagmus were calculated after each irrigation session. The Jongkees formula was used to determine canal paresis, which was considered pathologic when it was 20% or more. SOT was conducted by means of a dynamic posturography (Equitest System; NeuroCom International, Inc., Clackamas, OR, USA). The SOT evaluates postural stability with systematic changes in visual and somatosensory information available to the patient and determines which sensory inputs (vestibular, visual, or somatosensory) the patient relies upon most to maintain postural stability. In SOT, a vestibular ratio (ratio of mean values condition 5/condition 1) and a composite score (a weighted average of the scores from the six conditions of the SOT) were analyzed in patients. They were considered abnormal, when lower than the age-specific normative data given by the CDP manufacturer.

Vestibular-evoked myogenic potentials were recorded in the sitting position, with the head rotated away from the stimulated side during recording. The surface electrodes were placed as follows: the active electrode was placed over the middle third of the sternocleidomastoid muscle, the reference electrode was placed on the upper sternum, and the ground electrode was placed on the forehead. VEMPs were elicited using 500-Hz Blackman tone pips with a 2-ms rise/fall time and 1-ms plateau presented at a rate of 9/s through insert earphones. The stimulus intensities were 90-dB nHL, and the electromyography signal was amplified and bandpass filtered (30–1,500 Hz) using the GSI Audera system (Grason-Stadler, Eden Prairie, MN, USA). The results were considered pathological, if there was an interaural amplitude difference ratio greater than 40%, an interaural difference in threshold greater than 15 dB, or the absence of VEMP.

Video head impulse test (ICS Impulse, GN Otometrics, Taastrup, Denmark) was performed as described in a previous report (11). The vHIT gain was calculated as the ratio of the area under the curves of the eye to head movement, which was set by the device. A vHIT gain less than 0.8 in the lateral canal (LSCC) plane was considered abnormal. Gain asymmetry (GA) of the vHIT gains for head rotations to both sides were calculated using a general formula analogous to the formula for caloric canal paresis; GA = [(Gc − Gi)/(Gc + Gi)] × 100%, where Gc is the vHIT gain for head impulses exciting the contralateral canal and Gi is the vHIT gain for head impulses exciting the ipsilateral canal, which is the affected side. We defined abnormal GA ≥8.0% according to a previous study using normal subjects (14).

Our treatment protocol was as follows (13). Patients were advised to strictly adhere to a regular daily schedule with no caffeine and sufficient fluid intake. We also used a combination of Ginkgo biloba and antimigrainous preventive medications. The antimigrainous medications were administered using a three-step regimen. At the initial visit, the calcium channel blocker flunarizine (5 mg/day) was prescribed as a first-line medication. The effects of this drug were evaluated on the next follow-up visit, which was usually 2–3 weeks after the initial visit, and the next step [continuous use, increased dosage (10 mg/day), or medication switch] was determined by the extent of symptom improvement or presence of side effects. Patients were followed up on a monthly basis thereafter. Amitriptyline, a tricyclic amine, was used as a second-line medication if there was no improvement in symptoms or if side effects occurred with flunarizine. Amitriptyline was started at 10 mg/day, and the dosage was increased by 10–20 mg every month up to 90 mg/day until there was some improvement. Topiramate known as an anticonvulsant was administered as a third-line medication. The dosage started at 25 mg/day and was increased by 25 mg every week to a maximum of 100 mg/day. Topiramate was taken for more than 2 months.

The patients were assessed for symptomatic improvement 6 months after the initial visit. CR was defined as no need for continued medication, PR as improved symptoms but need for continued medication, and NR as no symptomatic improvement, thereby requiring a dose increase or medication change. We analyzed the relationships between responsiveness to these preventive medications and the results of the initial vestibular function tests.

All statistical analyses were conducted using SPSS 18.0 (SPSS software, SPSS Inc., Chicago, IL, USA). The chi-square test was used to compare the abnormal rates (patients with abnormal results/total) of vestibular function tests between the CR and combined PR plus NR groups. We also performed univariate logistic regression analyses to calculate the odds ratios and the corresponding 95% confidence intervals (CIs).

This study protocol was approved by the Institutional Review Board of the Asan Medical Center.

## Results

Eighty-one VM patients were enrolled in this study (19 males, 62 females; mean age 50.8 years, range 18–82 years; mean history of vertigo, 23 ± 34 months). There were no statistical differences in demographic features, including sex ratio and mean age, between the drug-responsive VM patient group (CR group) and partial/non-responsive group (PR plus NR) (Table 1).

**Table 1:** **Demographic characteristics of patients according to the drug treatment response at 6-month follow-up**.

Variables	No medication required at 6-month FU	Medication required at 6-month FU		*p*-value
	Complete recovery (*n* = 63)	Partial recovery (*n* = 7)	No recovery (*n* = 11)	
Sex, *n* (%)				0.496
Male	14 (74%)	1 (5%)	4 (21%)	
Female	49 (79%)	6 (10%)	7 (11%)	
Mean age (years)	51.4	48.9	49.7	0.899

*FU, follow-up; CR, complete recovery 6 months after the initial visit; PR, partial recovery 6 months after the initial visit; NR, no recovery 6 months after the initial visit*.

Table 2 shows the prevalence of each abnormal vestibular function test in both groups. At the initial evaluation, 9 of 81 patients (11%) exhibited abnormal vHIT results, 14 of 73 (19%) exhibited abnormal caloric test results, 25 of 65 (38%) exhibited abnormal SOT results, 8 of 75 (11%) exhibited abnormal cervical VEMP (cVEMP) results, and 20 of 75 (27%) exhibited abnormal ocular VEMP (oVEMP) results. The abnormal rates of the caloric test and vHIT gain significantly differed between groups (*p* < 0.05), while there was no significant group difference in the amplitude of catch-up saccades in vHIT, vestibular ratio in SOT, cVEMP responses, and oVEMP responses.

**Table 2:** **Results of vestibular function tests according to the drug treatment responses**.

Vestibular function test	No medication required at 6-month FU, *n* (%)	Medication required at 6-month FU, *n* (%)	*p*-value
**Caloric test**			
Normal (*n* = 59)	48 (81%)	11 (19%)	0.014
Abnormal (*n* = 14)	7 (50%)	7 (50%)	
**vHIT gain**			
Normal (*n* = 72)	59 (82%)	13 (18%)	0.011
Abnormal (*n* = 9)	4 (44%)	5 (56%)	
**CS amplitudes in vHIT**			
Normal (*n* = 72)	58 (80.6%)	14 (19.4%)	0.089
Abnormal (*n* = 9)	5 (55.6%)	4 (44.4%)	
**Vestibular ratio in SOT**			
Normal (*n* = 40)	30 (75%)	10 (25%)	0.539
Abnormal (*n* = 25)	17 (68%)	8 (32%)	
**cVEMP**			
Normal (*n* = 67)	50 (74.6%)	17 (25.4%)	0.420
Abnormal (*n* = 8)	7 (87.5%)	1 (12.5%)	
**oVEMP**			
Normal (*n* = 55)	40 (72.7%)	15 (27.3%)	0.271
Abnormal (*n* = 20)	17 (85%)	3 (15%)	

*FU, follow-up examination; CS, catch-up saccades; cVEMP, cervical vestibular-evoked myogenic potential; oVEMP, ocular vestibular-evoked myogenic potential; vHIT, video head impulse test; SOT, sensory organization test*.

Six months later, 63 of 81 patients (78%) no longer required medication (CR), while 7 exhibited PR and 11 exhibited NR. Prevalences of abnormal vHIT gain and caloric test results were significantly related to the necessity for continued medication at the 6-month follow-up (OR = 5.67 and 4.36, respectively, Table 3).

**Table 3:** **Prognostic factors affecting the necessity of medication in vestibular migraine patients**.

Variables	OR	95% CI for OR	
		Lower	Upper
Abnormal caloric test	4.364	1.268	15.013
Abnormal vHIT gain	5.673	1.337	24.075

*OR, odds ratio; vHIT, video head impulse test*.

## Discussion

Caloric stimulation is the most commonly used method to quantify the function of the lateral semicircular canals and identify the side of peripheral vestibular dysfunction. Our previous study revealed abnormal canal paresis based on caloric test results in 23% of VM patients (9). In the present study, 19% of VM patients exhibited abnormal caloric test results, within the range (8–25%) of previous studies (3, 7, 15, 16). vHIT assesses the function of the semicircular canals in response to high-frequency head movement, which is more suitable for assessing real-world vestibular function of the canals than the caloric test. Abnormal vHIT was found in approximately 10% of VM patients in other studies (10, 11), similar to our present results (11%). A few posturographic studies have assessed VM patients. While posturography is not sufficient to diagnose VM, it can detect balance problems in some patients with a normal vestibule-ocular reflex (17). In the present study, a substantial minority of VM patients (38.5%) had abnormal SOT results, in accordance with the 45% prevalence in our previous study (9). cVEMP and oVEMP tests assess the otolith organs (saccule and utricle). Decreased amplitudes in the cVEMP test have been reported in VM patients (8, 18, 19). In addition, abnormal VEMP findings, including latency prolongation and shift to a preferred frequency to 1 kHz, have been reported in VM patients (20). cVEMP has recently been proposed as a diagnostic tool for differentiating Meniere’s disease patients from VM patients using the 500-Hz asymmetry ratio and 1,000/500-Hz frequency ratio (12).

What is the clinical significance of vestibular abnormalities in VM patients? In our study, neither cVEMP nor oVEMP could predict the drug response of VM patients. Similarly, SOT failed to predict the drug response of VM patients. Alternatively, vHIT and caloric test results were predictive of medication response. The vast majority (81%) of patients with normal caloric test results at the initial evaluation were complete responders, compared with only 50% of patients with abnormal caloric test results. Similarly, complete recovery was observed in 82% of patients with normal vHIT gain, which was significantly higher than that observed in patients with abnormal vHIT gain (44% patients). These statistically significant differences suggest that vestibular abnormalities are closely related to the medication responsiveness of VM patients. In VM patients with recurrent vertigo attacks for more than 6 months, poor response of vertigo symptoms to medication was related to the abnormal vestibular ratio of SOT (*p* = 0.011) (13). In this same study, there was a tendency for poor drug responsiveness in VM patients with abnormal caloric results (*p* = 0.055) (13).

There is no proven pathophysiology of VM, and the diagnosis of VM is based on clusters of specific symptoms and subject to speculation. Several hypotheses have been proposed to explain how migraine can occur with audiovestibular symptoms. VM may result from spreading depression affecting the brainstem (3). Alternatively, vasospasm of the internal auditory artery could lead to peripheral vestibular dysfunction and VM (21). Further, audiovestibular symptoms may arise by trigeminal neurogenic inflammation in the labyrinth, resulting in local plasma extravasation, or through vasospasm of the internal auditory artery (22). It was reported that the lifetime prevalence of migraine was higher in Meniere’s disease group (56%) compared to controls (25%), arguing a pathophysiologic link between the two diseases (23). Thus, the balance of evidence appears to support contributions of both peripheral and central vestibular deficits in VM pathogenesis. However, it may be argued that a vestibular abnormality is a coincidental finding, and it is possible that some patients with vestibular abnormalities that have been classified as VM may in fact have a different disorder. It has been reported that comorbid conditions (Meniere’s disease, benign paroxysmal positional vertigo, or chronic subjective dizziness) are important contributors to vestibular symptoms and that induced vertigo can act as a migraine trigger (16, 24, 25). The current definition of VM might include migraine that is coincided with or caused by vestibular disorders, which cannot be well categorized so far.

## Conclusion

Abnormal results of vHIT and caloric tests, revealing semicircular canal dysfunction in VM patients, significantly reduced the probability of CR to preventive medications after 6 months of treatment, suggesting that peripheral vestibular abnormalities are closely related to the development of vertigo in VM patients.

## Author Contributions

WK and HP designed the study, collected the data, interpreted the data, drafted the manuscript, revised the manuscript, and approved the final version of the manuscript. SL and CY performed statistical analysis, interpreted the data, drafted the manuscript, and approved the final version of the manuscript. JA and JC collected the data, interpreted the data, drafted the manuscript, revised the manuscript, and approved the final version of the manuscript.

## Conflict of Interest Statement

The authors declare that the research was conducted in the absence of any commercial or financial relationships that could be construed as a potential conflict of interest.
